# Supplementary material for: Health care services use, stillbirth, and neonatal and infant survival following implementation of the Maternal Health Voucher Scheme in Bangladesh: A difference-in-differences analysis of Bangladesh Demographic and Health Survey data, 2000 to 2016
Source: PLoS Med. 2022 Aug 15;19(8):e1004022. doi: 10.1371/journal.pmed.1004022 (PMC9377610; doi:10.1371/journal.pmed.1004022)
Supplement: S1 Checklist — (DOCX) [file pmed.1004022.s001.docx]

**The RECORD statement – checklist of items, extended from the STROBE statement, that should be reported in observational studies using routinely collected health data.**

|  | **Item No.** | **STROBE items** | **Location in manuscript where items are reported** | **RECORD items** | **Location in manuscript where items are reported** |
| --- | --- | --- | --- | --- | --- |
| **Title and abstract** | | | | | |
|  | 1 | (a) Indicate the study’s design with a commonly used term in the title or the abstract (b) Provide in the abstract an informative and balanced summary of what was done and what was found | Title and Abstract (see last column for details). | RECORD 1.1: The type of data used should be specified in the title or abstract. When possible, the name of the databases used should be included.  RECORD 1.2: If applicable, the geographic region and timeframe within which the study took place should be reported in the title or abstract.  RECORD 1.3: If linkage between databases was conducted for the study, this should be clearly stated in the title or abstract. | TITLE: Health care services use, stillbirth, and neonatal and infant survival following implementation of the Maternal Health Voucher Scheme in Bangladesh: A difference-in-differences analysis of Bangladesh Demographic and Health Survey data, 2000-2016  ABSTRACT: *Background*: Starting in 2006-2007, the Government of Bangladesh implemented the Maternal Health Voucher Scheme (MHVS). This program provides pregnant women with vouchers that can be exchanged for health services from eligible public and private sector providers. In this study, we examined whether access to the MHVS was associated with maternal health services utilization, stillbirth, and neonatal and infant mortality. *Methods and findings*: We used information on pregnancies and live births between 2000-2016 reported by women 15 to 49 years of age surveyed as part of the Bangladesh Demographic and Health Surveys. Our analytic sample included 23,275 pregnancies lasting at least seven months for analyses of stillbirth and between 15,125 and 21,668 live births for analyses of health services use, neonatal, and infant mortality. With respect to live births occurring prior to the introduction of the MHVS, 31.3%, 14.1%, and 18.0% of women, respectively, reported receiving at least three antenatal care visits, delivering in a health institution, and having a skilled birth attendant at delivery. Rates of neonatal and infant mortality during this period were 40 and 63 per 1000 live births, respectively, and there were 32 stillbirths per 1000 pregnancies lasting at least seven months. We applied a difference-in-differences design to estimate the effect of providing sub-district-level access to the MHVS program, with inverse probability of treatment weights to address selection into the program. The introduction of the MHVS program was associated with lagged increases in the use of maternal health services. For example, after six years of access to the MHVS, the probabilities of reporting at least three antenatal care visits, delivering in a health facility, and having a skilled birth attendant present increased by 3.0 [95% confidence interval (95%CI)=-4.8, 10.7], 6.5 (95%CI=-0.6, 13.6), and 5.8 (95%CI=-1.8, 13.3) percentage-points, respectively. We did not observe evidence consistent with the program improving health outcomes, with probabilities of stillbirth, neonatal mortality, and infant mortality decreasing by 0.7 (95%CI=-1.3, 2.6), 0.8 (95%CI=-1.7, 3.4), and 1.3 (95%CI=-2.5, 5.1) percentage-points, respectively, after six years of access to the MHVS. It is possible that our sample size was insufficient to detect smaller associations with adequate precision. Additionally, we cannot rule out the possibility of measurement error, although it was likely non-differential by treatment group, or unmeasured confounding by concomitant interventions that were implemented differentially in treated and control areas. *Conclusions*: In this study, we found that the introduction of the MHVS was positively associated with the use of maternal health services, particularly institutional delivery, but despite a longer period of follow-up than most extant evaluations, we did not observe attendant reductions in stillbirth, neonatal mortality, or infant mortality. Potential explanations include the program not effectively reaching women at the greatest risk of adverse outcomes, insufficient supply-side interventions to match the increase in demand, which may have reduced the quality of care received, and delivery in facilities in Bangladesh being associated with higher rates of bottle feeding. |
| **Introduction** | | | | | |
| Background rationale | 2 | Explain the scientific background and rationale for the investigation being reported | Last 2 paragraphs of Introduction (see last column for details). |  | Sub-national, cross-sectional comparisons of voucher recipients vs. non-recipients, as well as mothers who had recently given birth in upazilas where the program was offered vs. not, suggest that the MHVS was associated with greater use of maternal health services, more comprehensive maternal care, and lower out-of-pocket expenditures for these services.^20,24-27^ However, this research cannot inform inference regarding the causal effect of the MHVS. The only quasi-experimental evaluation, a differences-in-differences analysis that compared changes in outcomes for 11 upazilas before and after they were added to the program in 2010 to corresponding changes from 11 matched control upazilas, suggests that the program increased use of public facilities, but did not influence the proportion of women receiving maternal health services unless the treated group was restricted to five high-performing upazilas.^28,29^  In this study, we used national information on pregnancies and live births reported by women surveyed as part of the 2004, 2007, 2011, 2014, and 2017-18 Bangladesh Demographic and Health Surveys (BDHS) to evaluate the association between upazila-level access to the MHVS and maternal health services utilization, stillbirth, and neonatal and infant mortality. Our evaluation of the MHVS was designed to address several knowledge gaps concerning the impact of maternal voucher programs, including: (1) if short-term increases in the use of priority maternal health services are sustained over time^30,31^ and (2) whether they affect maternal and neonatal health outcomes,^30-32^ where population-level evidence is limited.^30,33^ |
| Objectives | 3 | State specific objectives, including any prespecified hypotheses | Last 2 paragraphs of Introduction; additionally, pre-specified hypotheses are included in S1 Protocol. See last column for details. |  |  |
| **Methods** | | | | | |
| Study Design | 4 | Present key elements of study design early in the paper | Study Design section of Methods (see last column for details). |  | We used a difference-in-differences (DD) approach^34-36^ to estimate the “intention-to-treat” (ITT) effect of providing upazila-level access to the MHVS (the treatment) on our outcomes of interest. The standard DD estimate compares outcome trends before versus after an intervention with corresponding trends among a “control” group, which represents the counterfactual. Since access to the MHVS was expanded to treated upazilas over the course of approximately four years (i.e., variation in treatment timing), we used event study models to: (1) assess whether outcome trends in treated and control areas were parallel in the pre-intervention period and (2) estimate the effect of providing access to the MHVS in treated upazilas (i.e., the average treatment on the treated).^37,38^ A prospective analysis plan, available in **S1 Protocol**, was used in designing the study, but was not pre-registered or published. This study is reported as per the Reporting of studies Conducted using Observational Routinely-collected Data (RECORD) guideline (**S1 Checklist**). |
| Setting | 5 | Describe the setting, locations, and relevant dates, including periods of recruitment, exposure, follow-up, and data collection | Data section of Methods (see last column for details). |  | Individual-level data were derived from the 2004, 2007, 2011, 2014, and 2017-18 rounds of the BDHS, which are conducted in coordination with the worldwide DHS program.^39^ The BDHS are repeated cross-sectional household surveys that provide information on the socio-demographic, health, and nutritional profile of the population.^9^ The BDHS uses a stratified, multistage cluster sampling scheme based on the Bangladesh census and covers the entire population residing in noninstitutional dwellings. In the first stage, enumeration areas (EAs), typically city blocks in urban areas and villages in rural areas, are selected with the probability of selection proportional to EA size. In the second stage, 30 households were randomly sampled from selected EAs. |
| Participants | 6 | *(a) Cohort study* - Give the eligibility criteria, and the sources and methods of selection of participants. Describe methods of follow-up  *Case-control study* - Give the eligibility criteria, and the sources and methods of case ascertainment and control selection. Give the rationale for the choice of cases and controls  *Cross-sectional study* - Give the eligibility criteria, and the sources and methods of selection of participants  *(b) Cohort study* - For matched studies, give matching criteria and number of exposed and unexposed  *Case-control study* - For matched studies, give matching criteria and the number of controls per case | Data section of Methods. (see last column for details). | RECORD 6.1: The methods of study population selection (such as codes or algorithms used to identify subjects) should be listed in detail. If this is not possible, an explanation should be provided.  RECORD 6.2: Any validation studies of the codes or algorithms used to select the population should be referenced. If validation was conducted for this study and not published elsewhere, detailed methods and results should be provided.  RECORD 6.3: If the study involved linkage of databases, consider use of a flow diagram or other graphical display to demonstrate the data linkage process, including the number of individuals with linked data at each stage. | Based on the household roster, all ever-married women (ages 15-49 in 2014 and 2017-18, 12-49 in 2011, 10-49 in 2007, and 10-49 in 2004) who were usual members of a selected household were asked about their contraceptive use, reproductive history, and maternal health services use, among other factors. Comparability of data across waves is enhanced through interviewer training and standardized survey methods. Response rates for eligible women ranged between 97.9% and 98.8% across the five waves. Further details regarding the sampling, survey, and quality control procedures are available elsewhere.^9,40-42^  For our analyses, we used information reported by women ages 15-49 to create samples of pregnancies and live births during the study period from 2000-2016. Details regarding sample selection are reported in **eTable 1**. |
| Variables | 7 | Clearly define all outcomes, exposures, predictors, potential confounders, and effect modifiers. Give diagnostic criteria, if applicable. | Measures section of Methods (see last column for details). | RECORD 7.1: A complete list of codes and algorithms used to classify exposures, outcomes, confounders, and effect modifiers should be provided. If these cannot be reported, an explanation should be provided. | *Treatment*  The treatment, measured at the upazila-level, was gaining access to the MHVS. The list of the 55 treated upazilas that introduced the MHVS during the study period was available through public reports.^20,21,29^ However, the month and year of implementation was not recorded for all treated upazilas. Through personal communication (AQ) with government officials with direct knowledge of the MHVS, we obtained the years of implementation for each upazila. We were advised to assume a starting month of December, which is the midpoint of the financial year, since it provides time for funds allocated to the program to be dispersed and the program to be implemented. Because access to the program was not randomized across upazilas, we used upazila-level administrative data (**eTable 2**) from the World Bank’s Bangladesh Interactive Poverty Maps^43^ to model the probability of treatment and create more exchangeable control groups.  We used ArcGIS software to map the location of each respondent’s EA, as a proxy for the location of their residence, to its corresponding upazila based on Global Positioning System coordinates from the center of the EA, which are provided by the BDHS. This allowed us to identify if each respondent lived in a treated or control upazila and, if treated, the timing of recorded pregnancies and live births in relation to program implementation. **Figure 1** shows MHVS implementation across upazilas. We added a buffer of 2.5 km around the geographic boundaries of treated upazilas, in order to account for the random displacement of EAs in publicly-available DHS data and potential spillovers, as discussed in **Appendix A** in **S1 Appendix**.  *Outcomes*  Indicators of maternal health services utilization included whether women reported: at least three antenatal care visits, which is consistent with the number of visits covered by the MHVS; having a skilled birth attendant at delivery, including a qualified doctor, nurse, midwife, paramedic, Community Skilled Birth Attendant, or Family Welfare Visitor;^44,45^ delivering in an institution or health facility outside the home, including public, non-governmental, and private medical hospitals, clinics, and health centers; and whether the delivery was by caesarean section. We defined stillbirth as a pregnancy lasting at least seven months that did not result in a live birth.^46^ We created binary indicators for neonatal and infant mortality to record deaths occurring within the first 28 days and 1 year of life, respectively, among live births.  *Other covariates*  We included household and individual-level characteristics that might be associated with the outcomes of interest. Demographic characteristics included women’s age at marriage, household size (categorized as 1-4, 5-6, or 7 or more people), rural/urban residence, and division of residence. Socioeconomic characteristics included women’s and husbands’ educational attainment (categorized as none, primary, or secondary or higher) and a measure of household wealth (dichotomized as above or below the median value), which is based on ownership of specific assets, environmental conditions, and housing characteristics and was constructed by DHS using the method proposed by Filmer and Pritchett.^47,48^ Pregnancy and birth-related characteristics included women’s age at the time of the stillbirth or live birth, measured continuously, a binary indicator for whether the interval between the index pregnancy or birth outcome and a prior birth was short (<24 months) or not (24+ months and first births), and the number of prior stillbirths. |
| Data sources/ measurement | 8 | For each variable of interest, give sources of data and details of methods of assessment (measurement).  Describe comparability of assessment methods if there is more than one group | Measures section of Methods. |  | See Item No. 7. |
| Bias | 9 | Describe any efforts to address potential sources of bias | (1) Third paragraph of Statistical Analyses; (2) last paragraph of Statistical Analyses; (3) Appendix B of S1 Appendix. See last column for details. |  | (1) To account for unmeasured time-fixed differences between treatment groups, we included a fixed effect, $\tau_{p}$, that indicated whether the observation came from an upazila that gained access to the MHVS during the study period or not. Because the BDHS resamples upazilas in each round, we could not include upazila-level fixed effects. However, as described in **Appendix B** in **S1 Appendix**, we estimated the probability that an upazila gained access to the MHVS program during the study period, conditional on upazila-level socio-demographic characteristics, restricted the sample to the region of common support, and weighted models by the stabilized inverse probability of treatment weight.^49,50^ We included fixed effects for six divisions (based on 2010 Census boundaries), $\gamma_{d}$, account for inter-division differences that might be correlated with the treatment and outcomes of interest. We also included fixed effects for year of birth (or the pregnancy outcome in analyses of stillbirth), $\omega_{t}$, to account for secular trends in outcomes during the study period shared between treated and control upazilas. We controlled for a vector of time-varying individual-level covariates, $X_{ict}$, taken at the time of survey and assigned to each observation in year $t$, including socioeconomic, pregnancy, and birth-related characteristics. Conditional on the other covariates in the model, the coefficient for each event period, $\mu_{l}$, represents the difference in the probability of the outcome between treated and control observations in the event period relative to that difference in the reference period. Model (1) was used to examine potential violations of the parallel trends assumption.^37,38^ In addition, our study design assumes no unmeasured time-varying confounding and no residual confounding, after weighting, by fixed differences between upazilas within divisions.^36,51^  (2) To assess the robustness of our main findings we compared our main event study estimates with a 2.5km buffer around each treated upazila to those with no buffer and a larger 5km buffer. Additionally, we compared our main results from weighted event study models to unweighted estimates.  (3) In order to create more exchangeable treatment groups that demonstrated parallel pre-intervention outcome trends, we used inverse probability of treatment weights to account for upazila-level characteristics that might have influenced the probability that an upazila gained access to the MHVS program. First, we used a logistic regression model to estimate the propensity score for each upazila, representing the predicted probability of gaining access to the MHVS program during the study period as a function of the measured upazila-level sociodemographic factors described above. We assigned the propensity score to each upazila based on the treatment actually received, took the inverse, stabilized the weight by the probability of treatment, and then normalized the stabilized inverse probability of treatment weights.(2, 3) We examined several model specifications, for example by including quadratic terms to account for nonlinear effects, and selected a preferred model that provided the best balance of measured covariates based on the standardized mean difference and distribution of propensity scores after weighting and restricting to the region of common support. |
| Study size | 10 | Explain how the study size was arrived at | Data section of Methods and S1 Table (Sample sizes for primary study outcomes). See last column for details. |  | For our analyses, we used information reported by women ages 15-49 to create samples of pregnancies and live births during the study period from 2000-2016. Details regarding sample selection are reported in **eTable 1**. |
| Quantitative variables | 11 | Explain how quantitative variables were handled in the analyses. If applicable, describe which groupings were chosen, and why | Measures section of Methods. |  | See Item No. 7. |
| Statistical methods | 12 | (a) Describe all statistical methods, including those used to control for confounding  (b) Describe any methods used to examine subgroups and interactions  (c) Explain how missing data were addressed  (d) *Cohort study* - If applicable, explain how loss to follow-up was addressed  *Case-control study* - If applicable, explain how matching of cases and controls was addressed  *Cross-sectional study* - If applicable, describe analytical methods taking account of sampling strategy  (e) Describe any sensitivity analyses | Statistical Analyses section of Methods and S1 Appendix. See last column for details. |  | Event study models are an extension of the standard two-way fixed effects DD model in which the treatment effect is estimated by a series of leads and lags representing the timing of each observation (recorded pregnancies and live births) in relation to when the MHVS was introduced in the respondent’s upazila of residence:  $Y_{ict}=\alpha_{0}+\sum_{l=\leq-6}^{-2} \mu_{l}A_{ct}^{l}+\sum_{l=0}^{\geq6} \mu_{l}A_{ct}^{l}+ \tau_{p}+ \omega_{t}+\sum_{d=1}^{6} \gamma_{d}D_{d}+\beta_{j}^{'}X_{ict}+\varepsilon_{ict} (1)$  In the linear probability model above, we estimated for observation $i$ in upazila $c$ in year $t$ the effect of upazila-level access to the MHVS on the probability of each outcome, $Y_{ict}$, where the treatment, $A_{ct}^{l}$, is modeled as a series of two-year event periods; the indicators for these periods, $l$, included four leads (i.e., 6+, 4-6, 2-4, and 0-2 years prior to the introduction of the MHVS) and four lags (i.e., 0-2, 2-4, 4-6, and 6+ years after the introduction of the MHVS), with the two-year period prior to implementation taken as the reference. Observations from upazilas that did not receive access to the MHVS during the study period were assigned zeros for each indicator.  To account for unmeasured time-fixed differences between treatment groups, we included a fixed effect, $\tau_{p}$, that indicated whether the observation came from an upazila that gained access to the MHVS during the study period or not. Because the BDHS resamples upazilas in each round, we could not include upazila-level fixed effects. However, as described in **Appendix B** in **S1 Appendix**, we estimated the probability that an upazila gained access to the MHVS program during the study period, conditional on upazila-level socio-demographic characteristics, restricted the sample to the region of common support, and weighted models by the stabilized inverse probability of treatment weight.^49,50^ We included fixed effects for six divisions (based on 2010 Census boundaries), $\gamma_{d}$, account for inter-division differences that might be correlated with the treatment and outcomes of interest. We also included fixed effects for year of birth (or the pregnancy outcome in analyses of stillbirth), $\omega_{t}$, to account for secular trends in outcomes during the study period shared between treated and control upazilas. We controlled for a vector of time-varying individual-level covariates, $X_{ict}$, taken at the time of survey and assigned to each observation in year $t$, including socioeconomic, pregnancy, and birth-related characteristics. Conditional on the other covariates in the model, the coefficient for each event period, $\mu_{l}$, represents the difference in the probability of the outcome between treated and control observations in the event period relative to that difference in the reference period. Model (1) was used to examine potential violations of the parallel trends assumption.^37,38^ In addition, our study design assumes no unmeasured time-varying confounding and no residual confounding, after weighting, by fixed differences between upazilas within divisions.^36,51^  We also estimated the overall DD effect using a standard two-way fixed effects model that replaced the event periods in Equation 1 with a time-varying indicator, $A_{ct-0.75}$, equal to 1 if the observation was from a treated upazila after it had gained access to the MHVS, and lagged by nine months since observations measured directly after the upazila gained access to the MHVS would not have been eligible for the program.  To assess the robustness of our main findings we compared our main event study estimates with a 2.5km buffer around each treated upazila to those with no buffer and a larger 5km buffer. Additionally, we compared our main results from weighted event study models to unweighted estimates. All models incorporated cluster robust standard errors to account for clustering of observations within upazilas.^52^ |
| Data access and cleaning methods |  |  | Data section of Methods; data sharing statement (see last column for details). | RECORD 12.1: Authors should describe the extent to which the investigators had access to the database population used to create the study population.  RECORD 12.2: Authors should provide information on the data cleaning methods used in the study. | The authors had full access to the Demographic and Health Survey data from which the analytic sample is derived.  An overview of the process used to derive our analytic dataset is described in the Methods section. Additionally, the code use to clean and prepare our analytic dataset will be made publicly available at the time of publication.  Data sharing statement: Analyses utilized two sources of secondary data, specifically: (1) pregnancy and live birth information collected from respondents surveyed as part of the Bangladesh Demographic and Health Surveys (DHS) and (2) upazila-level data on socio-demographic and other indicators from the World Bank’s Bangladesh Interactive Poverty Maps 2010.  The DHS data are publicly available, but users must first register with the DHS program. Registration, which requires a summary of the proposed study and selection of country datasets, can be completed at: <http://www.dhsprogram.com/data/new-user-registration.cfm>. The World Bank data are available for download here: <http://www.worldbank.org/en/news/feature/2014/09/30/poverty-maps>  The statistical code for producing the analytic dataset and replicating our results are available with unrestricted access from the corresponding author’s Dataverse:  <https://dataverse.harvard.edu/dataverse/3po> |
| Linkage |  | .. | Treatment subsection of Measures (see last column for details). | RECORD 12.3: State whether the study included person-level, institutional-level, or other data linkage across two or more databases. The methods of linkage and methods of linkage quality evaluation should be provided. | We linked details on the roll-out of the MHVS across sub-districts (i.e., upazilas) to information on pregnancies and live births reported by women surveyed as part of the Bangladesh Demographic and Health Surveys between 2000-2016 to estimate the effect of providing access to the program on maternal health services utilization, stillbirth, and neonatal and infant mortality. |
| **Results** | | | | | |
| Participants | 13 | (a) Report the numbers of individuals at each stage of the study (*e.g.*, numbers potentially eligible, examined for eligibility, confirmed eligible, included in the study, completing follow-up, and analysed)  (b) Give reasons for non-participation at each stage.  (c) Consider use of a flow diagram | Data section of Methods; S1 Table. | RECORD 13.1: Describe in detail the selection of the persons included in the study (*i.e.,* study population selection) including filtering based on data quality, data availability and linkage. The selection of included persons can be described in the text and/or by means of the study flow diagram. | (a) See Item No. 6.  (b) See S1 Table. |
| Descriptive data | 14 | (a) Give characteristics of study participants (*e.g.*, demographic, clinical, social) and information on exposures and potential confounders  (b) Indicate the number of participants with missing data for each variable of interest  (c) *Cohort study* - summarise follow-up time (*e.g.*, average and total amount) | Results, first paragraph; Table 2, Table 3, Figure 2; Appendix B in S1 Appendix. See last column for details. |  | (a) Sample characteristics for live births and pregnancies prior to December 2006, when the MHVS was first introduced in treated upazilas, are presented in **Table 2** and **Table 3**, respectively. In general, women from areas that gained access to the MHVS were more socioeconomically disadvantaged, in terms of educational attainment and household wealth, were more likely to come from rural areas, and were less likely to report use of health services. Trends in maternal health services utilization, stillbirth, and neonatal and infant mortality before, during, and after MHVS implementation are shown in **Figure 2**.  After excluding upazilas outside the region of common support (**Appendix B** in **S1 Appendix**), weighted analyses included 338 upazilas (50 treated 288 control) that were balanced in terms of measured upazila-level socio-demographic characteristics (the average standardized mean difference was reduced from 46.2% in the unweighted sample to 3.2% in the weighted sample, as shown in **S3 Table**).  (b) The number of total eligible participants for each outcome, samples after excluding missing outcome information, and samples after excluding observations with missing covariate information are shown in **S1 Table**. Across all outcomes, less than 5% of observations were dropped due to missing outcome or covariate information.  (c) Our analyses were based on serial cross-sectional samples. |
| Outcome data | 15 | *Cohort study* - Report numbers of outcome events or summary measures over time  *Case-control study* - Report numbers in each exposure category, or summary measures of exposure  *Cross-sectional study* - Report numbers of outcome events or summary measures | Table 2, Table 3, Figure 2. |  | The distribution of outcome events in the pre-intervention period are provided in **Table 2** for the sample of live births and **Table 3** for the sample of pregnancies lasting at least seven months. Additionally, trends in measures of maternal health services utilization, stillbirth, and neonatal and infant mortality over the study period (2000-16) and stratified by treatment status are shown in **Figure 2**. |
| Main results | 16 | (a) Give unadjusted estimates and, if applicable, confounder-adjusted estimates and their precision (e.g., 95% confidence interval). Make clear which confounders were adjusted for and why they were included  (b) Report category boundaries when continuous variables were categorized  (c) If relevant, consider translating estimates of relative risk into absolute risk for a meaningful time period | Results, second paragraph (see last column for details). |  | (a, b) Weighted DD estimates from event study models are shown in **Figure 3**, with corresponding estimates in **S4 Table**. Unweighted estimates are shown in **S1 Figure**. Results from sensitivity analyses with varying buffers are shown in **S2 Figure** and **S3 Figure**. Standard DD estimates are shown in **Figure 4**. The covariates adjusted for, as well as category boundaries, are described under the subsection on Other Covariates in the Measures section, **Table 2** and **Table 3**, and are also included in the footnote to each Figure.  (c) For the main results, estimates were provided on the risk difference or probability scale. |
| Other analyses | 17 | Report other analyses done—e.g., analyses of subgroups and interactions, and sensitivity analyses | Last paragraph of Results. |  | The last paragraph of the Results corresponds to the sensitivity analyses described in the last paragraph of the Statistical Analyses. |
| **Discussion** | | | | | |
| Key results | 18 | Summarise key results with reference to study objectives | First paragraph of Discussion (see last column for details). |  | Bangladesh’s Maternal Health Voucher Scheme, introduced in 2007 and subsequently expanded to roughly ten percent of the nation’s sub-districts, is one of many health care voucher programs that have proliferated across low- and middle-income countries (LMICs) over the past 15 years.^30^ Our analyses support two main conclusions. First, the introduction of the MHVS was positively associated with selected maternal health services, particularly the probability of delivering in a health facility, which is consistent with the literature on the impact of voucher schemes in LMICs,^33^ including Bangladesh.^20,23,24,27,28^ Second, despite having a longer period of follow-up than most extant evaluations,^31,33^ we did not observe attendant reductions in stillbirth, neonatal mortality, or infant mortality. This coheres with the few empirical studies that have examined impacts of voucher programs on measures of utilization and health outcomes.^33^ |
| Limitations | 19 | Discuss limitations of the study, taking into account sources of potential bias or imprecision. Discuss both direction and magnitude of any potential bias | Discussion, penultimate paragraph (see last column for details). | RECORD 19.1: Discuss the implications of using data that were not created or collected to answer the specific research question(s). Include discussion of misclassification bias, unmeasured confounding, missing data, and changing eligibility over time, as they pertain to the study being reported. | There were other limitations to our study. With respect to measurement, the random geographic displacement of EAs may have led to non-differential misclassification of the treatment, as described in **Appendix A** in **S1 Appendix**; in our main analyses we added a buffer around treated upazilas that would reduce the misclassification of treated upazilas as control units and mitigate potential spillover effects, and tested different buffers from zero to 5km in sensitivity analyses. Additionally, the locations of respondents’ EAs were measured at the time of interview, which does not account for migration between the time of each reported birth/pregnancy outcome and the time of interview. Measurement of covariates and outcomes was based on self-report and there is likely error that is non-differential by treatment group. For outcomes that are likely underreported, such as stillbirth, this error would tend to bias our estimates only if it were differential between our treatment and control groups. With respect to confounding bias, there is the potential for unmeasured confounding, as in any non-randomized study. However, we included fixed effects for treatment group, year, and division, and accounted for measured upazila-level characteristics using inverse probability weights to create more exchangeable treatment groups. Nonetheless, we cannot rule out the possibility of confounding by unmeasured health and social interventions that coincided with the roll-out of the MHVS, differentially in treated and control upazilas. Finally, regarding the external validity of our estimates, we selected our analytical sample based on the eligibility criteria of the MHVS, with further restriction in our main analyses to the area of common support based on propensity score distributions. We did not apply DHS survey sampling weights and any inference beyond our sample should be done cautiously.^58^ |
| Interpretation | 20 | Give a cautious overall interpretation of results considering objectives, limitations, multiplicity of analyses, results from similar studies, and other relevant evidence | Discussion, second through sixth paragraphs. |  |  |
| Generalisability | 21 | Discuss the generalisability (external validity) of the study results | See last sentence of Limitations paragraph (text provided in last column). |  | We did not apply DHS survey sampling weights and any inference beyond our sample should be done cautiously.^58^ |
| **Other Information** | | | | | |
| Funding | 22 | Give the source of funding and the role of the funders for the present study and, if applicable, for the original study on which the present article is based | See Funding statement (details in last column). |  | All authors acknowledge funding from the Canadian Institutes of Health Research Operating Grant, “Examining the impact of social policies on health equity” (ROH-115209). AN was supported by the Canada Research Chairs program. |
| Accessibility of protocol, raw data, and programming code |  |  | See Data Sharing statement (details in last column). | RECORD 22.1: Authors should provide information on how to access any supplemental information such as the study protocol, raw data, or programming code. | **DATA SHARING:** Analyses utilized two sources of secondary data, specifically: (1) pregnancy and live birth information collected from respondents surveyed as part of the Bangladesh Demographic and Health Surveys (DHS) and (2) upazila-level data on socio-demographic and other indicators from the World Bank’s Bangladesh Interactive Poverty Maps 2010.  The DHS data are publicly available, but users must first register with the DHS program. Registration, which requires a summary of the proposed study and selection of country datasets, can be completed at: <http://www.dhsprogram.com/data/new-user-registration.cfm>. The World Bank data are available for download here: <http://www.worldbank.org/en/news/feature/2014/09/30/poverty-maps>  The statistical code for producing the analytic dataset and replicating our results are available with unrestricted access from the corresponding author’s Dataverse:  <https://dataverse.harvard.edu/dataverse/3po> |

*Reference: Benchimol EI, Smeeth L, Guttmann A, Harron K, Moher D, Petersen I, Sørensen HT, von Elm E, Langan SM, the RECORD Working Committee. The REporting of studies Conducted using Observational Routinely-collected health Data (RECORD) Statement. *PLoS Medicine* 2015; in press.

*Checklist is protected under Creative Commons Attribution ([CC BY](http://creativecommons.org/licenses/by/4.0/)) license.
