# Supplementary material for: Health care services use, stillbirth, and neonatal and infant survival following implementation of the Maternal Health Voucher Scheme in Bangladesh: A difference-in-differences analysis of Bangladesh Demographic and Health Survey data, 2000 to 2016
Source: PLoS Med. 2022 Aug 15;19(8):e1004022. doi: 10.1371/journal.pmed.1004022 (PMC9377610; doi:10.1371/journal.pmed.1004022)
Supplement: S1 Protocol — (PDF) [file pmed.1004022.s002.pdf]

STUDY OVERVIEW

1. Principal Investigator(s)

Arijit Nandi
Thomas J Charters
Oduro Oppong-Nkrumah
Amm Quamruzzaman

2. Title

Impacts of the demand-side financing Maternal Health Voucher Scheme in Bangladesh on maternal and child health

3. Abstract

The government of Bangladesh implemented the Maternal Health Voucher Scheme (MHVS) in 2007 to reduce demand-side barriers to maternal health services in poorer populations. Using birth histories collected from repeated cross-sections of the Bangladesh Demographic and Health Survey (BDHS) we will estimate the effect of the MHVS on measures of maternal health service utilization (i.e., receipt of antenatal care, postnatal care use, delivery assisted by a health professional, deliveries conducted at a health facility, and use of Caesarian section) and neonatal and infant mortality using a difference-in-differences design. This approach compares trends in maternal and child health outcomes among BDHS respondents living near sub-districts (upazilas) targeted by the voucher scheme between 2007 and 2014 with corresponding trends among residents that lacked geographic access to the program. Further, we will assess whether the program affected socioeconomic inequalities in our primary outcomes.

INTERVENTION OVERVIEW

4. Intervention

The demand-side financing Maternal Health Voucher Scheme (MHVS) was implemented in 2007 with the intention of reducing financial barriers to accessing maternal care among poorer households and thereby increasing utilization of care. Specific services covered by vouchers include: up to three antenatal care (ANC) checkups; safe facility delivery or home delivery by a skilled birth attendant; a single postnatal care (PNC) checkup up to 6 weeks post-delivery; expenses for transport to ANC, delivery or PNC services; and costs associated with pregnancy complications (i.e., Caesarean-section). Additionally, pregnant women who deliver at facilities receive a cash amount for purchase of nutritious food and a baby-care gift box. At its inception, the MHVS program was either universal (in 9 upazilas) or means-tested (i.e., targeted households below the poverty line) depending on subdistrict of residence and was available for women in their first or second pregnancies; since then, all subdistricts adopted means-tested eligibility requirements (personal communication, Amm Quamruzzaman). In 2016 the program enrolled more than 107,000 beneficiaries in 53 upazilas. While previous research found increases in health services use attributable to the program, less is known about its long-run effects following the pilot phase or impacts on infant health.

5. Implementing Agency

The MHVS is implemented by the Bangladesh Ministry of Health and Family Welfare (MOHFW) with support from the World Health Organization (WHO) in areas of technical and financial support.

6. Intervention Timeline
The intervention began in 2007, was subsequently expanded to new areas, and is currently ongoing.

7. Start Date
The intervention was first implemented as a pilot program in selected disadvantaged subdistricts (upazilas) based on their literacy rate, population density, poverty level, and presence of community based skilled birth attendants (CBSA). Although the program was officially launched in July 2004, implementation was delayed until 2006, The most recent additions to the program in 2017-18 will not be included in the treated group because they were added after the end of the study period.

8. End Date
Ongoing program.

EVALUATION METHOD OVERVIEW

9. Primary (or First) Evaluation Method
Difference-in-differences

10. Details of Evaluation Approach
We will use a difference-in-differences approach to compare trends in maternal and child health outcomes between 2007 and 2014 among BDHS respondents living near sub-districts (upazilas) targeted by the voucher scheme (the "treated" group) with corresponding trends among residents that lacked geographic access to the program (the "control" group). This approach assumes that the control group accurately substitutes for the counterfactual, specifically, the trends in maternal and child health outcomes in treated upazilas had they not received access to the MHVS program. Although this assumption cannot be empirically verified, we will examine whether pre-intervention trends in our primary outcomes for the treated and control groups are parallel, both visually and statistically. If trends appear non-parallel in the pre-intervention period, we will consider the use of matching to identify a subset of upazilas from the control group that more accurately mimic outcome trends in treated areas. Additionally, we must assume no unmeasured time-varying confounding as well as no differences in fixed characteristics that might induce differential outcome trends for the treated and control groups. We will conduct literature reviews and interviews with officials familiar with the program to attempt to identify any concurrent interventions or other factors (time-varying confounders) that may have influenced our primary outcomes of interest. We will estimate the average treatment effect on the treated, which represents the effect of the MHVS program on maternal and child health outcomes in areas that were targeted by the program. Further, we will assess whether the program affected socioeconomic inequalities in our primary outcomes, based on maternal education or household asset index.

11. Outcomes (Endpoints)

Our analysis will investigate the impact of the MHVS on intermediate and final outcomes. Intermediate outcomes will include measures of maternal health services utilization for that were explicitly funded by the MHVS, specifically antenatal care, postnatal care, delivery assisted by a health professional, deliveries conducted at a health facility, and use of Caesarian section. This information is collected by BDHS respondents for live births occurring in the five years prior to each survey. An additional outcome might include contraception use, as stakeholders expressed concern that the MHVS would disincentivize use in poorer populations. Final outcomes will address infant health and include risk of neonatal and infant mortality.

12. Hypotheses

We hypothesize that indicators of maternal health services utilization improved to a greater extent in areas that gained access to the MHVS than in other areas that did not, given that these were the immediate targets of the program. Because the program was targeted based on levels of household poverty, the impacts should be greater for more socioeconomically disadvantaged groups. Whether the program translated to improvements in infant survival is unclear, as this depends not only on the receipt of health services in the perinatal period, but also on the quality of services received, as well as other factors.

13. Unit of Intervention or Assignment

The intervention was assigned at the upazila level.

14. Unit of Analysis

We will collect information on live births occurring from 1999 to 2014 occurring to respondents of the BDHS, including information on health services used and infant survival.

15. Number of Clusters in Sample

For the purposes of our analyses, clusters are enumeration areas used by the BDHS as primary sampling units. Respondents living in enumeration areas near at least one of the 53 treated upazilas will represent the treatment group whereas respondents from enumeration areas farther (e.g., <10 km) from treated upazilas will define the control group.

16. Number of Individuals in Sample

The sample is developed through aggregated counts from the reports of a total of 69,572 women of reproductive age accounting for a total of 85,180 births over the entire study period and 4,875 deaths of children prior to reaching one year of age.

17. Size of Treatment, Control, or Comparison Subsamples *

The exact sample size is to be determined, although we expect to draw information from roughly 75,000 live births to women surveyed as part of the BDHS.

Bangladesh		No. Women Interviewed		No. births		No. Infant Deaths
1997-2006		27619		63890		3985
2007-2014		41953		21290		890
Total		69572		85180		4875

The count of women interviewed refers to the number of respondents in the DHS survey being women of reproductive age 15-49. The number of births refers to the numbers of births reported by the respondent women from the pre-intervention period 1997-2006 and the post-intervention period 2007-2014. The number of infant deaths is a subset of children born who died prior to 1 year of age.

METHODS

18. Description of sample

The Bangladesh Demographic and Health Surveys (BDHS) are nationally representative household surveys providing information on indicators of population health and nutrition. The standard BDHS is cross-sectional and collects data every few years to permit comparison of estimates. The BDHS uses a multistage cluster sampling scheme based on the Bangladesh census and covers the entire population residing in private dwellings. Information on live births is collected from ever-married women ages 15-49 at the time of interview. Further details regarding the sampling and survey procedures are available in the BDHS technical documentation.

19. Key Data Sources

We will use data collected from the Bangladesh Standard Demographic and Health Surveys (BDHS) collected in the years 1999-2000, 2004, 2007, 2011, and 2014. Note that GPS coordinates do not seem available as part of the 1996-1997 BDHS.

20. Statistical Analyses

We will compare pre-treatment trends in primary outcomes for treated versus control groups and examine the statistical evidence for non-parallel trends. If trends appear non-parallel in the pre-intervention period, we will consider the use of matching or weighting to identify a group of upazilas from the control group that more accurately mimic outcome trends in treated areas. We will estimate the impact of the MHVS using a logistic model for our binary outcomes, with post-estimation to produce estimates on the additive/absolute scale, of the general form:

$$logit(Y_{ict}) = \beta_0 + \beta_1 T_{ct-1} + \tau_c + \omega_t + \beta_j' X_{ict} + \beta_k' Z_{ct},$$

where Y_{ict} is the outcome related to birth i in cluster c in year t; T_{ct-1} is a time-varying indicator equal to 1 if the household is located in an enumeration area near a treated upazila (treated) and 0 otherwise (control), lagged by one year to respect the temporality between the intervention and the measurement of the outcome; τ_c is a fixed effect for treatment group that accounts for unmeasured time-fixed differences between treated and control areas; ω_t are fixed effects for birth years to account for shared secular trends in primary outcomes during the study period; X_{ict} is a vector of time-varying individual-level covariates taken at the time of the DHS survey and assigned to each live birth in year t; and Z_{ct-1} is a vector of census enumeration level characteristics taken in the year prior to the measurement of the outcome. The model will be extended to examine effect measure modification by socioeconomic status (SES), specifically maternal education and household wealth, by including cross-products between these socioeconomic characteristics and T_{ct-1}, as well as between SES and τ_c, and SES and ω_t. In sensitivity analyses we will: (i) examine evidence

for lead and lagged effects and (2) vary the distance thresholds used to distinguish households in treated versus control clusters. All analyses will account for spatial autocorrelation (i.e., clustering of observations within upazilas) in the estimation of standard errors using a spatial Durbin model.

DISCUSSION AND THREATS TO VALIDITY

21. Internal validity

Presence of co-interventions impacting our outcomes at similar times to the introduction of the MHVS may confound our effect measures and threaten internal validity. The MHVS took place in the context of a number of health sector reforms and non-governmental initiatives progressing towards Millennium Development Goals pertinent to under-five and maternal mortality. However, the MHVS was not implemented concurrently with an expansion in supply-side services. Some complementary initiatives such as promotion of community based skilled birth attendants (beginning 2001) took place prior to the MHVS. Other initiatives such as the Perinatal Care Project (participatory women's groups) and Improved Neonatal Maternal Child Survival program (IMNCS: community health workers) were implemented all or predominantly in the period following the MHVS and shown to impact several of our outcomes related to health services use. However, the scope of these programs appears limited to a series of highly localized settings and likely impacted a comparatively much smaller number of participatory households. In other instances government supply-side reforms and NGO programs may be thought of complementary to the MHVS. Other threats to internal validity include the potential for selection bias, since we are observing outcomes only for live births and, in some cases (i.e., infant mortality), only for live births that survived to a certain age.

22. External validity

The MHVS was enacted in response to failures of supply-side reforms in Bangladesh to increase use of maternal care in poorer populations. The program's focus on reducing demand-side barriers to health services access was specific to Bangladesh and in line with a highly focused reform strategy centering on women's health and gender equity. Although we anticipate the findings of our research will be valid in regard to Bangladesh, caution should be taken in applying the findings to different country settings.
