# Supplementary material for: Health care services use, stillbirth, and neonatal and infant survival following implementation of the Maternal Health Voucher Scheme in Bangladesh: A difference-in-differences analysis of Bangladesh Demographic and Health Survey data, 2000 to 2016
Source: PLoS Med. 2022 Aug 15;19(8):e1004022. doi: 10.1371/journal.pmed.1004022 (PMC9377610; doi:10.1371/journal.pmed.1004022)
Supplement: S1 Appendix — (DOCX) [file pmed.1004022.s003.docx]

**APPENDIX A.** Mapping of enumeration areas (EAs) to treated or control upazilas.

Mapping of EAs to either treated or control upazilas was done using ArcGIS software. First, we merged the shapefiles provided by the Demographic and Health Surveys, which provide the latitude and longitude coordinates for the EAs sampled in each survey year, to map the 2594 EAs that were included in the five survey waves (i.e., 2004, 2007, 2011, 2014, 2017-18). Second, we added an upazila boundaries shapefile (i.e., Sub-national Administrative Units of Bangladesh) available from [www.gadm.org](http://www.gadm.org), version 3.4, accessed in April 2018. Third, we created separate selections of the 55 treated upazilas, which gained access to the MHVS during the study period, and 490 control upazilas that did not. Fourth we performed a spatial join to identify treated EAs within treated upazilas. We also assigned control EAs (including control) to their upazila to merge upazila-level administrative data for other analyses (i.e., estimation of propensity scores).

The locations of EAs are randomly displaced by the DHS in order to protect respondent confidentiality; based on the 2011 BDHS, average displacement was roughly 1 km for urban EAs and 2 km in rural EAs, compared to a mean upazila area of 319km^2^.(1) Because this can result in the non-differential misclassification of the treatment (e.g., an EA just outside a treated upazila may have been randomly displaced within its boundaries and vice versa), and also to reduce potential spillovers (i.e., respondents in EAs just outside of a treated upazila utilizing the program), we added a buffer of 2.5km around the boundaries of treated upazilas. These results were also compared to those with no buffer and a larger buffer of 5km.

**APPENDIX B. Inverse probability of treatment weights**

Among the 513 upazilas represented in the Bangladesh Demographic and Health Survey waves, we were able to link 508 upazilas (52 treated and 456 control) to the administrative data sources described in **S2 Table**. As shown in **S3 Table**, treated upazilas that were more likely to gain access to the MHVS during the study period were socioeconomically disadvantaged compared to control areas that did not, with a lower percent of the population/households, on average, who were: literate; completed primary or greater schooling; above the lower or upper poverty lines; worked in industry or services; or from a household with electricity, a toilet, or tap water.

In order to create more exchangeable treatment groups that demonstrated parallel pre-intervention outcome trends, we used inverse probability of treatment weights to account for upazila-level characteristics that might have influenced the probability that an upazila gained access to the MHVS program (**S2 Table)** including: age structure, rural population, literacy, educational attainment, school attendance, poverty, employment, and household characteristics. First, we used a logistic regression model to estimate the propensity score for each upazila, representing the predicted probability of gaining access to the MHVS program during the study period as a function of the measured upazila-level sociodemographic factors described above. We assigned the propensity score to each upazila based on the treatment actually received, took the inverse, stabilized the weight by the probability of treatment, and then normalized the stabilized inverse probability of treatment weights.(2, 3) We examined several model specifications, for example by including quadratic terms to account for nonlinear effects, and selected a preferred model that provided the best balance of measured covariates based on the standardized mean difference and distribution of propensity scores after weighting and restricting to the region of common support. The preferred model included the following covariates: proportions of the total population 0-6, 7-14, and 65 and older (with adults 15-64 as the reference); literacy (i.e., proportion of adults who can write a letter); proportions of adults who completed primary education, secondary education, and university (with less than primary education as the reference); proportion attending school among those 6-18 years of age; proportions of population below the official national upper poverty line, the official national lower poverty line, and who belong to the bottom 40% of the national real per capita consumption distribution; share of the population who live in rural areas; proportions employed in services and industry, among those employed (with agricultural employment as the reference); proportions of households with access to electricity, with a flush toilet, with access to a non-flush latrine, without a toilet who practice open defecation, and with access to tube-well water; a quadratic for literacy; a quadratic for employment in industry.

Based on the distribution of propensity scores (shown below), we excluded upazilas outside the region of common support, primarily control upazilas that were unlikely to gain access to the MHVS based on their socio-demographic profiles, and the sample was reduced to 338 upazilas (50 treated 288 control). As shown in **S3 Table**, the weighted distributions of socio-demographic characteristics were similar for the treated and control groups of upazilas. The average standardized mean difference was reduced from 46.2% in the unweighted sample to 3.2% in the weighted sample, with values lower than 10% for all covariates. The stabilized inverse probability of treatment weights after restricting to the region of common support were distributed with a mean and median of 1.01, and a range from 0.22 to 2.00.

**
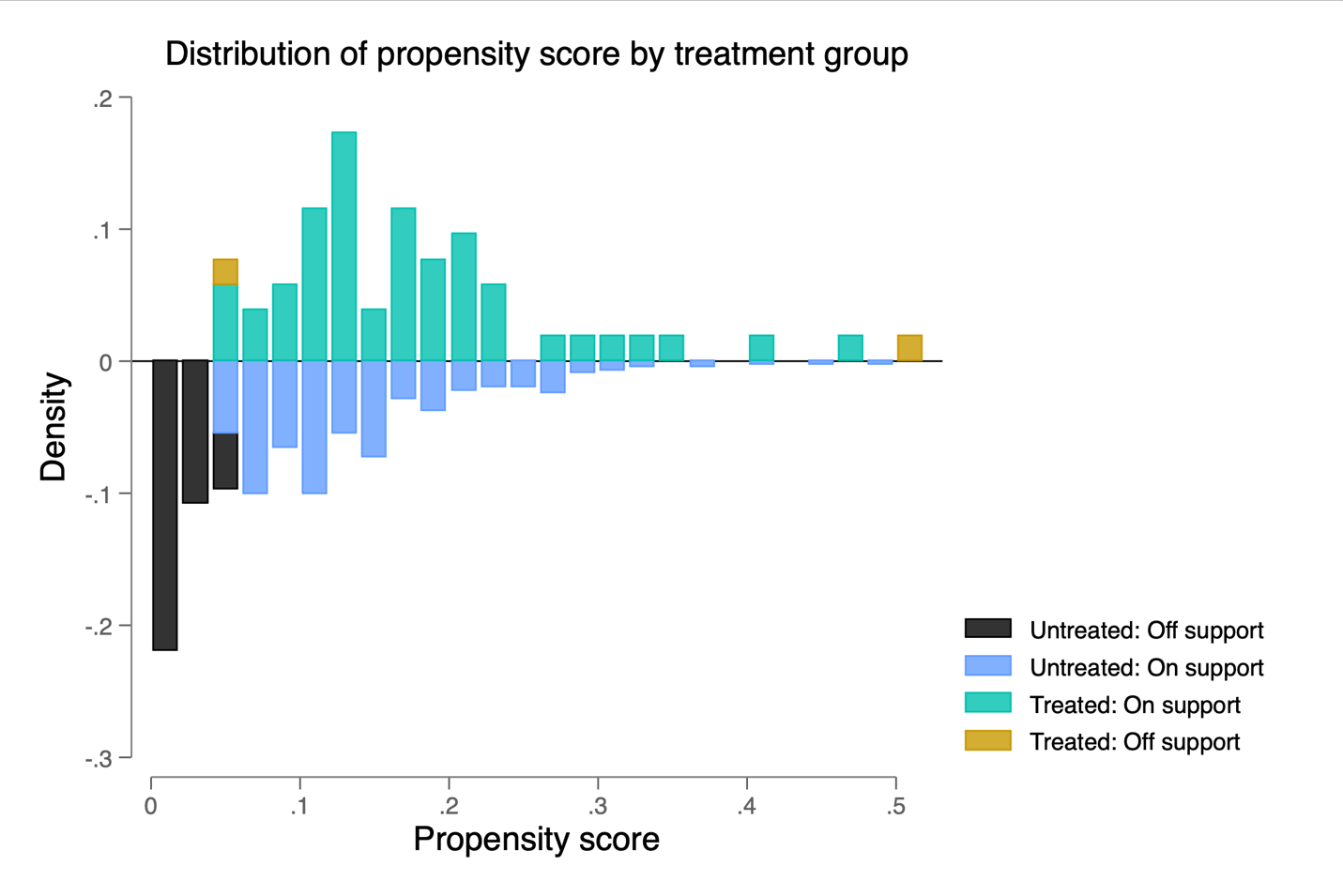
**

**APPENDIX C. Results for delivery by caesarean section**

As shown in the Figure below, rates of caesarean delivery increased markedly over the study period (panel A), as was the case for the other health services related outcomes evaluated.

*Trends in caesarean delivery among Bangladesh Maternal Health Voucher Scheme respondents, 2000-16*


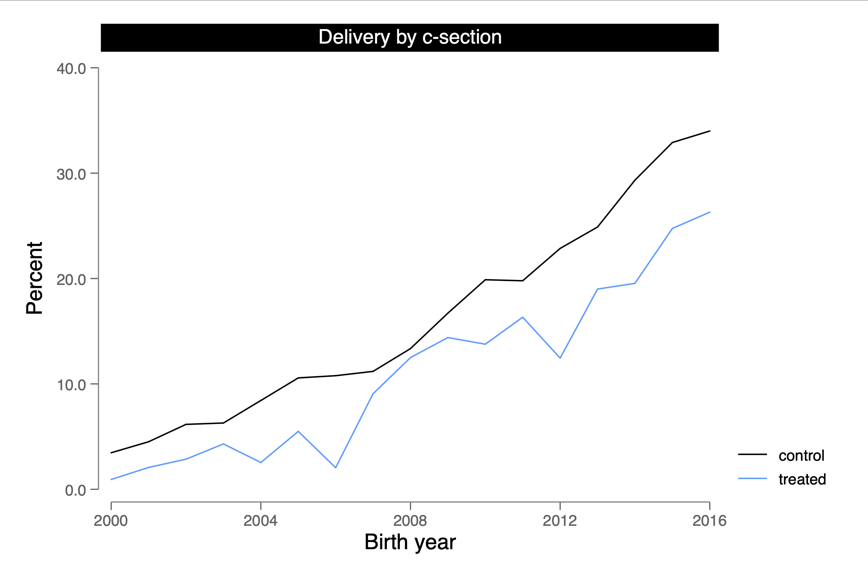


However, event study models showed that control upazilas did not represent a valid substitute for the counterfactual trend, given evidence of diverging pre-intervention trends between treated and control groups in both unweighted and weighted analyses. As such, the effects of the MHVS on caesarean delivery were not estimated.

*Event study estimates of the effect of gaining access to the Maternal Health Voucher Scheme in specified periods before and after the implementation of the program on the probability of caesarean delivery; Bangladesh Demographic and Health Survey Data, 2000-16*

*
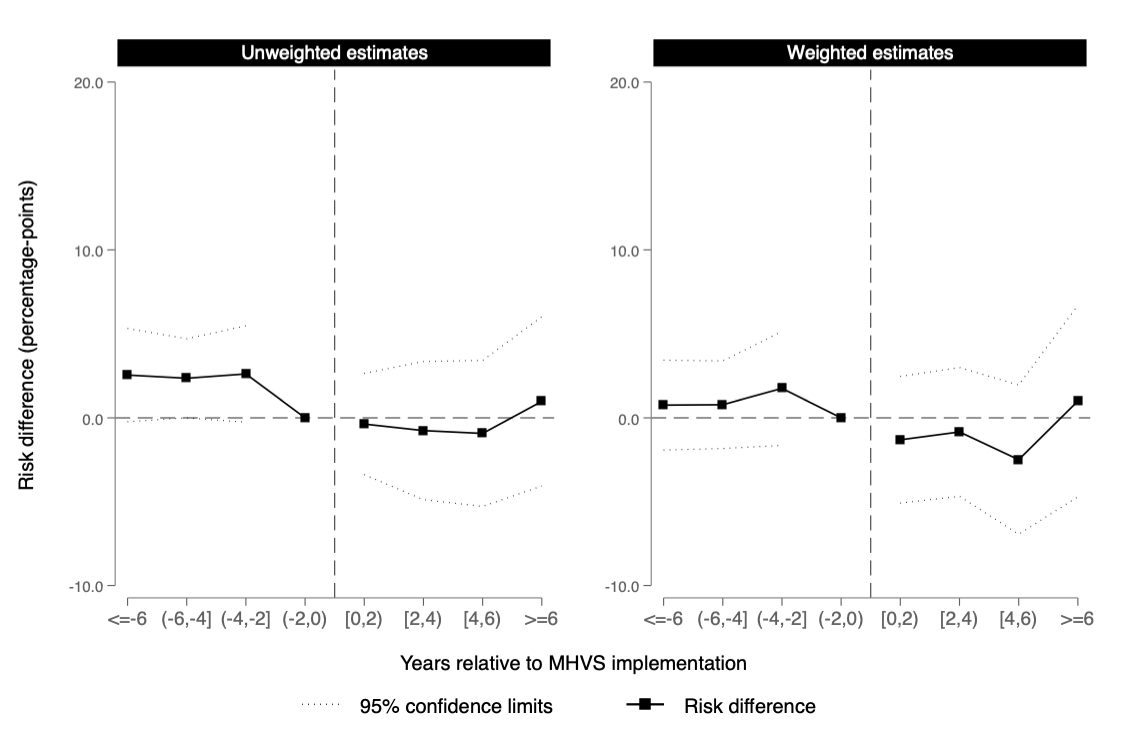
*

*Notes:* Models included fixed effects for six divisions, fixed effects for year of birth (or the pregnancy outcome in analyses of stillbirth), and a vector of time-varying individual-level covariates, including household size, maternal age at marriage, women’s age at the time of the stillbirth or live birth, rural residence, women’s and husband’s educational attainment, household wealth (above or below median), whether the interval between the index pregnancy or birth outcome and a prior birth was short (<24 months) or not (24+ months and first births), and prior stillbirth (in analyses of stillbirth). Weights accounted for the following upazila-level characteristics described in **S2 Table**: age structure, rural population, literacy, educational attainment, school attendance, poverty, employment, and household characteristics. Event periods included four leads (i.e., 6+, 4-6, 2-4, and 0-2 years prior to the introduction of the MHVS) and four lags (i.e., 0-2, 2-4, 4-6, and 6+ years after the introduction of the MHVS), with the two-year period prior to implementation taken as the reference. Each coefficient represents the difference in the probability of the outcome (percentage-point differences) comparing treated and control observations in the event period relative to the reference period. 95% confidence intervals account for the clustering of observations within upazilas. Sample sizes are reported in **S1 Table,** and the estimation of weights is described in **Appendix B** in **S1 Appendix**.

**REFERENCES CITED**

1. Burgert C, Colston J, Roy T, Zachary B. Geographic displacement procedure and georeferenced data release policy for the Demographic and Health Surveys. DHS Spatial Analysis Reports No. 7. Calverton, Maryland, USA: ICF International; 2013.

2. Austin PC, Stuart EA. Moving towards best practice when using inverse probability of treatment weighting (IPTW) using the propensity score to estimate causal treatment effects in observational studies. Statistics in medicine 2015;34(28):3661-3679.

3. Garrido MM, Kelley AS, Paris J, Roza K, Meier DE, Morrison RS, et al. Methods for constructing and assessing propensity scores. Health services research 2014;49(5):1701-1720.
