# Supplementary material for: Health care services use, stillbirth, and neonatal and infant survival following implementation of the Maternal Health Voucher Scheme in Bangladesh: A difference-in-differences analysis of Bangladesh Demographic and Health Survey data, 2000 to 2016
Source: PLoS Med. 2022 Aug 15;19(8):e1004022. doi: 10.1371/journal.pmed.1004022 (PMC9377610; doi:10.1371/journal.pmed.1004022)
Supplement: S1 Table — (DOCX) [file pmed.1004022.s004.docx]

**S1 Table.** Sample sizes for primary study outcomes

|  | **Total eligible**^1^ | **After excluding missing outcome info** | **After excluding missing covariate data** | **Total on support**^5^ |
| --- | --- | --- | --- | --- |
| 3+ Antenatal care visits^2^ | 24699 | 24677 | 23947 | 15125 |
| Institutional delivery^2^ | 28897 | 28819 | 28024 | 17880 |
| Skilled birth attendant^2^ | 28897 | 28872 | 28074 | 17910 |
| Caesarean section^2^ | 28897 | 28871 | 28072 | 17910 |
| Stillbirth^3^ | 37909 | 37909 | 36677 | 23275 |
| Neonatal mortality^4^ | 35231 | 35231 | 34094 | 21668 |
| Infant mortality^4^ | 29752 | 29752 | 28781 | 18268 |

^1^Total observations (live births or, in the case of stillbirth, pregnancies lasting at least 7 months) reported by women ages 15-49 years within the study period (2000-2016) after restricting to pregnancy and birth outcomes occurring in the 5 years prior to interview date

^2^For indicators of maternal health services use, information was available for all children born alive in the previous five years (past three years in 2014 and 2017 surveys), although information on antenatal care was only asked of the most recent birth during these reference periods.

^3^For the analysis for stillbirth, we used information collected in the monthly calendar of reproduction and contraceptive use in the past five years to identify pregnancies lasting at least seven months.

^4^For analyses of neonatal and infant mortality, we used information collected on the vital status of all live births in the past five years that occurred at least 28 days and at least one year prior to the DHS interview date, respectively, to ascertain whether each child survived the neonatal (28 day) and infant (one year) periods following birth.

^5^Total observations in the region of common support, based on the distribution of the propensity score for treated and control upazilas, used in weighted analyses.
