# Supplementary material for: Health care services use, stillbirth, and neonatal and infant survival following implementation of the Maternal Health Voucher Scheme in Bangladesh: A difference-in-differences analysis of Bangladesh Demographic and Health Survey data, 2000 to 2016
Source: PLoS Med. 2022 Aug 15;19(8):e1004022. doi: 10.1371/journal.pmed.1004022 (PMC9377610; doi:10.1371/journal.pmed.1004022)
Supplement: S2 Table — (DOCX) [file pmed.1004022.s005.docx]

**S2 Table.** Upazila-level characteristics from the World Bank’s Bangladesh Interactive Poverty Maps^1^

| Indicator | Definition |
| --- | --- |
| Age structure | Total population 0-6, 7-14, 15-64, and 65 and older |
| Rural population | Share of the upazila who live in rural areas |
| Literacy | Proportion of adults who can write a letter |
| Educational attainment | Proportion of adults who completed less than primary education |
|  | Proportion of adults who completed primary education |
|  | Proportion of adults who completed secondary education |
|  | Proportion of adults who have completed university |
| School attendance | Children 6-18 years old who attend school |
| Poverty | Proportion of population below the official national upper poverty line |
|  | Proportion of population below the official national lower poverty line |
|  | Proportion of population who belong to the bottom 40% of the national real per capita consumption distribution |
| Employment | If employed, sector of employment is agriculture |
|  | If employed, sector of employment is industry |
|  | If employed, sector of employment is services |
| Household characteristics | Percent of households with access to electricity |
|  | Percent of households with a flush toilet |
|  | Percent of households with access to a non-flush latrine |
|  | Percent of households without a toilet who practice open defecation |
|  | Percent of households with access to tap water |
|  | Percent of households with access to tube-well water |

^1^All indicators computed from the 2011 Census of Population and Housing available from the Integrated Public Use Microdata Series project (IPUMS), other than poverty indicators, which were from the 2010 Bangladesh Poverty Maps
