# Supplementary material for: Health care services use, stillbirth, and neonatal and infant survival following implementation of the Maternal Health Voucher Scheme in Bangladesh: A difference-in-differences analysis of Bangladesh Demographic and Health Survey data, 2000 to 2016
Source: PLoS Med. 2022 Aug 15;19(8):e1004022. doi: 10.1371/journal.pmed.1004022 (PMC9377610; doi:10.1371/journal.pmed.1004022)
Supplement: S3 Table — (DOCX) [file pmed.1004022.s006.docx]

**S3 Table.** Standardized mean differences comparing upazila-level sociodemographic factors in the unweighted and stabilized inverse probability of treatment weighted samples

|  | **Unweighted** | | | **Weighted** | | |
| --- | --- | --- | --- | --- | --- | --- |
| *Variable* | *Mean, Treated* | *Mean, Control* | *%bias* | *Mean, Treated* | *Mean, Control* | *%bias* |
| pop 0-6 years^1^ | 16.4 | 15.4 | 37.4 | 16.4 | 16.3 | 2.1 |
| pop 7-14 years^1^ | 20.0 | 19.1 | 38.6 | 19.8 | 19.9 | -3.5 |
| pop 15-64 years^1^ | 58.4 | 60.7 | -50 | 58.6 | 58.6 | 0.7 |
| pop 65+^1^ | 5.3 | 4.8 | 46.9 | 5.2 | 5.2 | -1.1 |
| adult literacy^2^ | 43.7 | 50.4 | -57.4 | 44.6 | 44.2 | 4.3 |
| <primary school^3^ | 57.9 | 50.5 | 64 | 56.8 | 57.1 | -3.8 |
| primary completed^3^ | 31.3 | 33.0 | -24.4 | 31.8 | 31.6 | 2.7 |
| secondary completed^3^ | 9.0 | 12.7 | -77.3 | 9.4 | 9.3 | 4.2 |
| university completed^3^ | 1.8 | 3.9 | -55.9 | 2.0 | 1.9 | 8.4 |
| school att 6-18 yrs^4^ | 69.7 | 70.3 | -8.2 | 70.6 | 70.3 | 3.7 |
| <upper pov line^5^ | 35.7 | 30.2 | 39 | 35.2 | 35.3 | -1.2 |
| <lower pov line^6^ | 21.3 | 17.1 | 38.9 | 20.9 | 21.0 | -0.7 |
| <40% PCC^7^ | 47.2 | 38.8 | 41.7 | 46.0 | 46.5 | -2.7 |
| rural pop^8^ | 89.9 | 75.0 | 69.5 | 89.3 | 89.4 | -1.6 |
| agriculture^9^ | 64.6 | 51.9 | 64.1 | 64.9 | 64.9 | -0.3 |
| industry^9^ | 7.7 | 11.8 | -51.5 | 8.0 | 7.8 | 4.7 |
| services^9^ | 27.7 | 36.3 | -56.9 | 27.1 | 27.3 | -1.6 |
| hh w/ electricity^10^ | 44.0 | 55.1 | -51.6 | 42.7 | 43.5 | -4.4 |
| hh w/ toilet | 18.7 | 26.1 | -51.5 | 20.3 | 19.7 | 5.3 |
| hh w/ latrine | 39.3 | 38.5 | 5.3 | 38.2 | 38.5 | -1.8 |
| hh w/o toilet | 9.5 | 7.3 | 23.9 | 8.7 | 9.3 | -5.5 |
| hh w/ tap water | 1.1 | 11.1 | -58.7 | 1.3 | 1.5 | -8.3 |
| hh w/ tube-well | 91.4 | 81.8 | 49.5 | 91.0 | 90.8 | 1.6 |
| **Mean bias** |  |  | **46.2** |  |  | **3.2** |

^1^Total population per age category; ^2^Proportion of adults who can write a letter; ^3^Adult educational attainment; ^4^Proportion children 6-18 years old who attend school; ^5^ Proportion of population below the official national upper poverty line; ^6^Proportion of population below the official national lower poverty line; ^7^ Proportion of population who belong to the bottom 40% of the national real per capita consumption distribution; ^8^ Share of the upazila who live in rural areas; ^9^ If employed, sector of employment; ^10^Percent of households with each characteristic
