# Supplementary material for: Health care services use, stillbirth, and neonatal and infant survival following implementation of the Maternal Health Voucher Scheme in Bangladesh: A difference-in-differences analysis of Bangladesh Demographic and Health Survey data, 2000 to 2016
Source: PLoS Med. 2022 Aug 15;19(8):e1004022. doi: 10.1371/journal.pmed.1004022 (PMC9377610; doi:10.1371/journal.pmed.1004022)
Supplement: S1 Fig — (DOCX) [file pmed.1004022.s008.docx]

**S1 Figure**. Unweighted event study estimates of the effect of gaining access to the Maternal Health Voucher Scheme in specified periods before and after the implementation of the program; Bangladesh Demographic and Health Survey Data, 2000-16
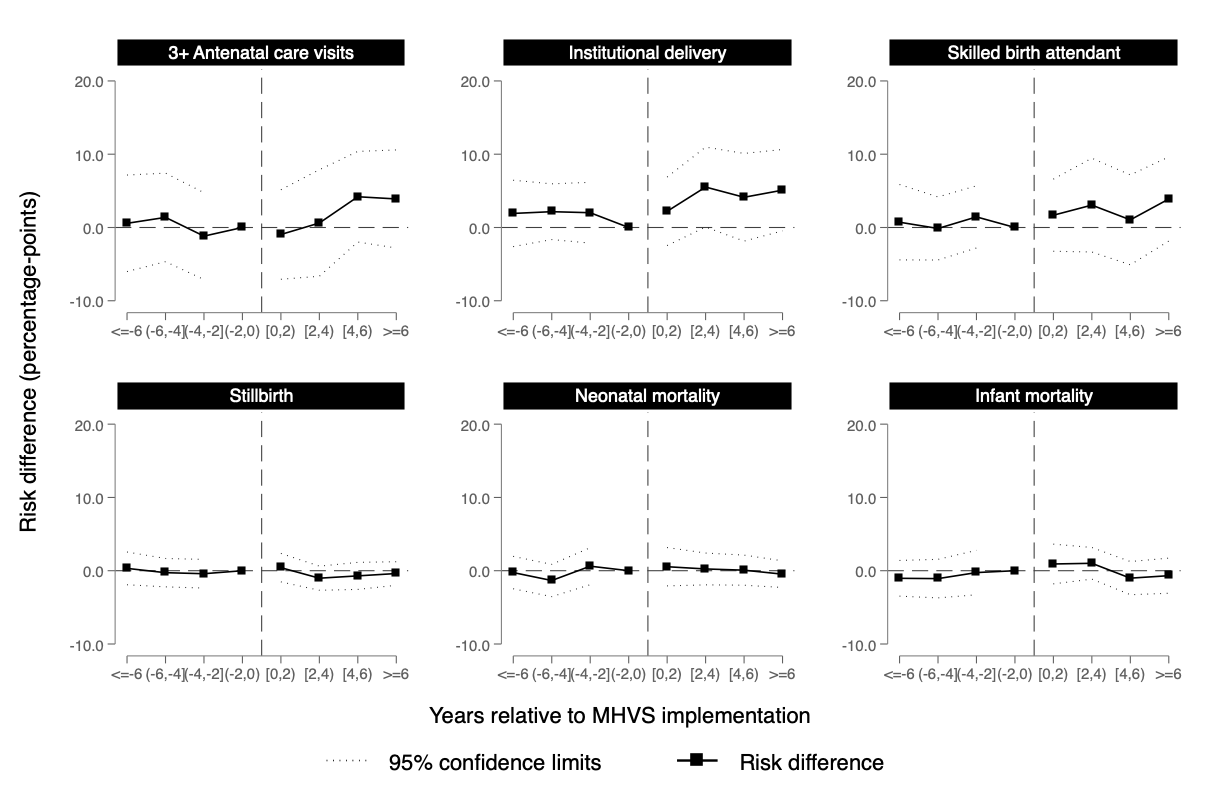


*Notes:* Models included fixed effects for six divisions, fixed effects for year of birth (or the pregnancy outcome in analyses of stillbirth), and a vector of time-varying individual-level covariates, including household size, maternal age at marriage, women’s age at the time of the stillbirth or live birth, rural residence, women’s and husband’s educational attainment, household wealth (above or below median), whether the interval between the index pregnancy or birth outcome and a prior birth was short (<24 months) or not (24+ months and first births), and prior stillbirth (in analyses of stillbirth). Event periods included four leads (i.e., 6+, 4-6, 2-4, and 0-2 years prior to the introduction of the MHVS) and four lags (i.e., 0-2, 2-4, 4-6, and 6+ years after the introduction of the MHVS), with the two-year period prior to implementation taken as the reference. Each coefficient represents the difference in the probability of the outcome (percentage-point differences) comparing treated and control observations in the event period relative to the reference period. 95% confidence intervals account for the clustering of observations within upazilas. Sample sizes are reported in **S1 Table**.
