# Supplementary material for: Health care services use, stillbirth, and neonatal and infant survival following implementation of the Maternal Health Voucher Scheme in Bangladesh: A difference-in-differences analysis of Bangladesh Demographic and Health Survey data, 2000 to 2016
Source: PLoS Med. 2022 Aug 15;19(8):e1004022. doi: 10.1371/journal.pmed.1004022 (PMC9377610; doi:10.1371/journal.pmed.1004022)
Supplement: Attachment — Submitted filename: response to reviewers 20220512.pdf [file pmed.1004022.s013.pdf]

McGill

<u>**REQUESTS FROM THE EDITORS:**</u>

Abstract Methods and Findings:
Line 69 - please revise to 'After six years of access…'

Thank you, this error has been corrected in the Abstract.

Abstract Conclusions:
Please briefly address the specific implications of your study, substantiated by the results.

We have added a concluding sentence on the implications of our findings, specifically "*Further work and engagement with stakeholders is needed to assess if the MHVS has affected the quality of care and health inequalities, and whether the design and eligibility of the program should be modified to improve maternal and neonatal health outcomes.*"

Introduction:
Line 167 - please clarify ' unlikely to reach socioeconomically disadvantaged groups absent efforts'

We have clarified this statement.

Methods:
Thank you for providing your prospective analysis plan and further details of changes to planned analyses in your cover letter. Please include these details of where reported analyses differed from those planned in the Methods section of the paper (including those made in response to peer review comments), with rationale.

We have added the following details to this section: "*A prospective analysis plan, available in **S1 Protocol**, was used in designing the study, but was not pre-registered or published. In contrast to the analysis plan, we added stillbirth as an outcome and did not evaluate whether the program has affected socioeconomic inequalities in our primary outcomes, due to inadequate sample sizes and the imprecision of stratified estimates. In addition, we incorporated event study models and inverse probability weights in our DD analyses, to assess evidence for parallel pre-intervention trends and construct more comparable treatment groups.*"

Results:
Line 387 - please clarify '3.5% among treated vs. 3.2% among treated'

Thank you for identifying this error. In the revised manuscript we have edited this to indicate that the 3.2% is among the control group.

Figures:
Please confirm that the GADM map is compatible with CC BY licensing

Confirmed. The GADM license (available here: https://gadm.org/license.html) indicates the following: "*You are allowed (but not required) to publish these articles (and the maps they contain) under an open license such as CC-BY as is the case with PLoS journals and may be the case with other open access articles.*"

Tables:
Table S4 - please define RD, UCL and LCL in the table legend.

We have added a superscript that defines the abbreviations used in Table S4.

References:
Your reference list appears to have been duplicated; please revise.

This has been corrected in the revised manuscript.

<u>COMMENTS FROM THE REVIEWERS:</u>

REVIEWER #1: ALEX MCCONNACHIE, STATISTICAL REVIEW

I thank the authors once again for considering my comments, and I am happy with this latest version. I have no further comments to make.

Thank you, Professor McConnachie. We very much appreciate your careful review and comments on our paper.

Reviewer #3:

This revised version is much improved and the inclusion of the BDHS 2017-18 data in my opinion strengthens the analysis significantly in terms of the key finding that there does NOT seem to be any impact of the Voucher Scheme on any of the key outcomes. Irrespective of any statistical nuancing, Figure 2 is very clear in terms of lack of any substantive difference between voucher scheme recipients and counterfactuals. The final version of the paper still talks about a notable lagged effect on improved facility births, which I frankly don't see. The authors may consider a more explicit recognition of this finding not being as robust as they make out.

We thank the Reviewer for their comments and for encouraging us to include the 2017-8 BDHS, in particular, which we agree adds to the strength of the paper. The purpose of Figure 2 is to present trends in our primary outcomes over the study period, but it should not be used to infer whether the program has had an effect, particularly since the program was phased in across treated upazilas between 2006 and the end of the study period (i.e., there is heterogeneity in

McGill

treatment timing). The event study model results shown in Figure 3 align observations based on the timing of implementation in the upazila of residence, and accounts for other sources of potential confounding, and are more appropriate for this purpose. This Figure, as well as the difference-in-differences estimates shown in Figure 4, indicate a lagged effect of program access on the most directly incentivized outcome—institutional delivery. We agree with the Reviewer that we did not find evidence consistent with the program having a positive impact on other outcomes, including stillbirth, neonatal mortality, and infant mortality.

If this analysis is published, as I hope it might given the importance of the findings, I would strongly recommend an accompanying commentary to discuss these counterintuitive findings and their implications for policy.

We agree! Further research and policy discourse is needed, as we noted in the revised Conclusions section of the Abstract.
