# Supplementary material for: Health care services use, stillbirth, and neonatal and infant survival following implementation of the Maternal Health Voucher Scheme in Bangladesh: A difference-in-differences analysis of Bangladesh Demographic and Health Survey data, 2000 to 2016
Source: PLoS Med. 2022 Aug 15;19(8):e1004022. doi: 10.1371/journal.pmed.1004022 (PMC9377610; doi:10.1371/journal.pmed.1004022)
Supplement: Attachment — Submitted filename: response to reviewers 20111117.docx [file pmed.1004022.s011.docx]

**REQUESTS FROM THE EDITORS:**

1. General comments
   a. Please include line numbers in your revised manuscript; preferably not starting from 1 with each new page.

**Line numbers (continuous) have been added to the revised manuscript.**

b. Please avoid inadvertently using causal language e.g. effect, effectiveness, impact, etc.

**We defer to the Editors and conventions of the journal and have revised the text throughout by removing causal language. In the revised manuscript, we use the term “effect” only when indicating that our objective was to evaluate or estimate the effect of the MHVS program, but refer to our estimates as associations.**

c. Throughout the paper, please adapt reference call-outs to the following style: "... under five and maternal mortality [1,3]." (noting the absence of spaces within the square brackets).

**We have adopted the style indicated.**

2. Title: Please revise your title according to PLOS Medicine's style. "Impact of" should be used only if causality can be inferred, i.e., from an RCT. Please place the study design in the subtitle (ie, after a colon). We suggest “Health care services use, stillbirth, and neonatal and infant survival following implementation of the Maternal Health Voucher Scheme in Bangladesh: A difference-indifferences analysis of Bangladesh Demographic and Health Survey data, 2000-2013” or similar

**We appreciate the suggestion and have adopted the recommended title.**

3. Abstract Background: The final sentence should clearly state the study question.

**In the revised manuscript, we have added the study question to the final sentence of the Abstract (Background): “In this study, we examined whether access to the MHVS was associated with maternal health services utilization, stillbirth, and neonatal and infant mortality.”**

4. Abstract Methods and Findings:
   a. Please provide brief demographic details of the study population

**We have added that the results are based on information reported by women ages 15-49 years.**

b. Please include the number of participants

**We have added the analytic sample sizes that correspond to the inverse probability weighted estimates presented in the abstract: “Our analytic sample included 18,154 pregnancies lasting at least seven months for analyses of stillbirth and between 12,844 and 17,253 live births for analyses of health services use, neonatal, and infant mortality.”**

c. Please report the main findings of your study, in keeping with the main outcome measures specified e.g. stillbirth, etc.

**We have revised the Abstract and now report findings for all main outcomes as follows: “We did not observe evidence consistent with the program improving health outcomes, with probabilities of stillbirth, neonatal mortality, and infant mortality decreasing by 1.6 (95%CI=-0.3, 3.4), 0.9 (95%CI=-1.5, 3.4), and 1.8 (95%CI=-1.3, 4.9) after four years of access to the MHVS.”**

d. Please include the actual numbers / probabilities, in addition to the differences reported.

**We have reported the pre-intervention/baseline percentages corresponding to each outcome: “With respect to live births occurring prior to the introduction of the MHVS, 31.3%, 14.1%, and 18.0% of women, respectively, reported receiving at least three antenatal care visits, delivering in a health institution, and having a skilled birth attendant at delivery. Rates of neonatal and infant mortality during this period were 40 and 63 per 1000 live births, respectively, and there were 32 stillbirths per 1000 pregnancies lasting at least seven months.”**

e. In the last sentence of the Abstract Methods and Findings section, please describe 2-3 of the main limitations of the study's methodology.

**We have added the study’s main limitations to the last sentence of the Methods and Findings section of the Abstract: “It is possible that our sample size was insufficient to detect smaller effects with adequate precision. Additionally, we cannot rule out the possibility of measurement error, although it was likely non-differential by treatment group, or unmeasured confounding by concomitant interventions that were implemented differentially in treated and control areas.”**

5. Abstract Conclusions: Please begin your Abstract Conclusions with "In this study, we observed ..." or similar, to summarize the main findings from your study, without overstating your conclusions. Please emphasize what is new and address the implications of your study, being careful to avoid assertions of primacy.

**We have revised the first sentence of the Abstract Conclusions as follows: “In this study, we found that the introduction of the MHVS was positively associated with the use of maternal health services, but despite a longer period of follow-up than most extant evaluations, we did not observe attendant reductions in stillbirth, neonatal mortality, or infant mortality.”**

6. Author Summary: At this stage, we ask that you include a short, non-technical Author Summary of your research to make findings accessible to a wide audience that includes both scientists and non-scientists. The Author Summary should immediately follow the Abstract in your revised manuscript. This text is subject to editorial change and should be distinct from the scientific abstract. Please see our author guidelines for more information: <https://journals.plos.org/plosmedicine/s/revising-your-manuscript#loc-author-summary> In the final bullet point of ‘What Do These Findings Mean?’, please describe the main limitations of the study in non-technical language.

**An Author Summary has been added to the revised manuscript.**

7. Introduction:
   a. Please address causal language in paragraph beginning ‘In this study…’

**We have revised the causal language at the beginning of this paragraph.**

b. Please remove subheadings within your Introduction

**The Introduction has been edited to remove subheadings (i.e., description of the MHVS).**

c. Please relocate the section beginning “The MHVS was implemented…” to precede the paragraph beginning “Sub-national, cross-sectional comparisons of voucher recipients vs. non-recipients, …”, followed finally by the paragraph beginning ‘In this study…” (which should the the final paragraph of your Introduction)

**Done.**

8. Methods:
   a. Did your study have a prospective protocol or analysis plan? Please state this (either way) early in the Methods section. If a prospective analysis plan (from your funding proposal, IRB or other ethics committee submission, study protocol, or other planning document written before analyzing the data) was used in designing the study, please include the relevant prospectively written document written before analyzing the data) was used in designing the study, please include the relevant prospectively written document with your revised manuscript as a Supporting Information file to be published alongside your study, and cite it in the Methods section. A legend for this file should be included at the end of your manuscript. If no such document exists, please make sure that the Methods section transparently describes when analyses were planned, and if/when reported analyses differed from those that were planned. Changes in the analysis-- including those made in response to peer review comments-- should be identified as such in the Methods section of the paper, with rationale. If a reported analysis was performed based on an interesting but unanticipated pattern in the data, please be clear that the analysis was data-driven.

**This study is part of a larger program of work, the** [**PROSPERED project**](https://www.prosperedproject.com/)**, with the objective of identifying health and social policy levers for achieving the health-related targets set forth by the United Nations Sustainable Development Goals. As is common with our project, we drafted a research protocol using a format adapted from 3ie’s Registry for International Development Impact Evaluations (RIDIE) that was circulated among co-authors, for the purposes of articulating research questions and hypotheses and pre-specifying our methodological approach and evaluation design. The most recent version on record is dated April 30^th^, 2019 (circulated to co-authors on May 1, 2019), which preceded the initiation of our statistical analyses. However, we did not pre-register or publish the protocol in a prospective registry, and therefore lack a date stamp, which we have acknowledged in the manuscript.**

**This protocol has been added as a Supporting Information file, S1 Protocol, and cited in the Methods section. Although we did not update the protocol while conducting the analyses, the approach presented in our paper is faithful to that specified in our protocol, in terms of our research question, hypotheses, and evaluation approach, with a few exceptions. Prior to any analyses: (1) we dropped postnatal care as an outcome because it was not measured consistently across waves of the Bangladesh Demographic and Health Surveys and (2) we added stillbirth as an outcome. During our data analyses: (1) we applied additional inclusion criteria, described in eTable 1, which lowered our expected sample size; (2) we lacked the precision to explore heterogeneity by socioeconomic status and did not include these subgroup analyses; and (3) we incorporated event study models, since we became aware of these methods for addressing heterogeneity in treatment timing; and (4) we added inverse probability of treatment weights (Appendix B in S1 Appendix) to create more exchangeable treatment groups while assessing evidence for parallel trends.**

b. Please provide more information regarding ethical approval of your study e.g. reference number

**We have added the reference number: “The Institutional Review Board of McGill University reviewed and approved this study** (**IRB Study Number A05-E42-17B)”**

c. Please ensure that the study is reported according to the RECORD guideline, and include the completed RECORD checklist as Supporting Information. Please add the following statement, or similar, to the Methods: "This study is reported as per the Reporting of studies Conducted using Observational Routinely-collected Data (RECORD) guideline (S1 Checklist)." The RECORD guideline can be found here: https://www.record-statement.org/ When completing the checklist, please use section and paragraph numbers, rather than page numbers which will likely no longer correspond to the appropriate sections after copy-editing.

**This statement has been added to the Methods, along with the completed RECORD checklist (S1 Checklist).**

d. Please note: Authors are responsible for keeping documentation of the nature and course of the personal communication with government officials.

**Thank you. We have maintained a record of personal communication with government officials regarding the implementation of the MHVS.**

9. Results:
   a. Please provide a single table showing the baseline characteristics of the study population.

**We have two different analytic samples defined by our outcomes: (1) live births for analyses of maternal health care services use and infant and neonatal mortality and (2) pregnancies lasting at least 7 months for analyses of stillbirth. Presently we report the baseline characteristics for live births and pregnancies in Tables 2 and 3, respectively, for the total sample, treated group, and control group. Combining these estimates into a single table will be challenging, given the number of columns and slight distinctions in the covariates (rows). Thus, our preference is to maintain the current presentation with two tables, but we defer to the Editor and would be happy to consider an alternative arrangement.**

b. Please quantify more of the results in the main text of your Results section.

**We have added comparisons for the treated and control groups in terms of their baseline (pre-intervention) distributions of the primary outcomes: “Women from treated areas were also less likely to report use of health services than women in control areas, including at least three antenatal care visits 22.2% vs. 32.8%), delivering in an institution or health facility (7.2% vs. 15.3%), and having a skilled birth attendant at delivery (10.8% vs. 19.1%). Treated and control groups were similar in their distributions of neonatal mortality (4.0% in both groups), infant mortality (5.7% among treated vs. 6.4% among control), and stillbirth (3.4% among treated vs. 3.2% among treated).”**

**Additionally, as in the abstract, we added difference-in-differences estimates for all outcomes, including stillbirth, neonatal mortality, and infant mortality: “We did not observe evidence consistent with the program having an impact on the probabilities of stillbirth, neonatal mortality, or infant mortality, with probabilities of stillbirth, neonatal mortality, and infant mortality decreasing by 1.6 (95%CI=-0.3, 3.4), 0.9 (95%CI=-1.5, 3.4), and 1.8 (95%CI=-1.3, 4.9) after four years of access to the MHVS.”**

c. Please provide the actual numbers of events for the outcomes where possible, in addition to summary statistics.

**We have added the frequencies and the actual numbers of events are provided in the corresponding Tables, which are cited in the Results section.**

d. For the probabilities reported in your results, please specify the comparison group.

**In the revised Results section, we have specified the comparison group for the weighted DD estimates.**

10. Discussion:
    a. Please present and organize the Discussion as follows: a short, clear summary of the article's findings; what the study adds to existing research and where and why the results may differ from previous research; strengths and limitations of the study; implications and next steps for research, clinical practice, and/or public policy; one-paragraph conclusion.

**We have adapted the Discussion so that it follows the recommended organization (i.e., initial summary paragraph, a second paragraph placing our results in context with existing literature, a substantive discussion of our main findings, strengths and limitations, and implications and conclusions).**

b. Please avoid the use of causal language

**We have revised the Discussion accordingly and avoided causal language when referring to our Results.**

11. Figures: Where weighted estimates are provided, please indicate which factors are adjusted for / taken into consideration, in the respective figure legend.

**Thanks for this suggestion. The factors accounted for, both through regression adjustment and weighting, have been added to the legends.**

12. Tables:
    a. Please define abbreviations used in the table legend of each table (including Supplementary files)

**Done.**

b. Where weighted estimates are provided, please indicate which factors are adjusted for / taken into consideration, in the respective figure legend.

**The factors accounted for have been added to the legends.**

c. Tables 2 & 3 - please separate columns ‘Total’ and ‘Control’. Please present numerators and denominators for percentages. Please clarify whether the results presented are indeed Mean and SD for characteristics such as Rural Residence, Mother’s Education, Birth Interval <24 months, etc, as % would make more sense in these (and other) instances.

**We have separated the columns and now report means and SDs for continuous factors and frequencies and percentages for categorical characteristics, as recommended by the Statistical Reviewer.**

13. References:
    a. Please ensure that journal name abbreviations match those found in the National Center for Biotechnology Information (NCBI) databases, and are appropriately formatted and capitalised.

**We have reviewed the abbreviated journal names and corrected issues with formatting and abbreviation.**

b. Please also see https://journals.plos.org/plosmedicine/s/submission-guidelines#loc-references for further details on reference formatting. For example, 6 authors should be cited prior to ‘et al’

**We have consulted the reference guide and corrected formatting errors.**

c. Where website addresses are cited, please specify the date of access.

**Access dates have been included.**

14. Supplementary files:
    a. Please provide titles and legends for each individual table and figure in the Supporting Information.

**Titles and legends for all supporting information has been added following the conventions cited below.**

b. Please see https://journals.plos.org/plosmedicine/s/supporting-information for our guidelines regarding file names.

**Done.**

**Comments from the reviewers:**

**Reviewer #1: Alex McConnachie, Statistical Review**

Nandi's paper looks at data from the Bangladesh Demographic and Health Surveys, to assess whether the introduction of the Nandi's paper looks at data from the Bangladesh Demographic and Health Surveys, to assess whether the introduction of the Maternal Health Voucher Scheme to some areas of the country had any impact on maternal health service use and outcomes. This review considers the use of statistics in the paper. The analysis methods are generally very good. The IPTW approach is appropriate for this analysis, and the model used to assess the lagged impact of the MHVS seems good.

**We appreciate the reviewer’s comments and positive assessment of our approach.**

1. The main conclusion is that the MHVS led to increased use of health services, but not health outcomes. Whilst I agree that this appears to be the trend, it is notable that none of the effect estimates reported are statistically significant, so I would question the strength of the evidence at this stage. Should the wording be a little more cautious?

**We agree that the confidence limits for the event study estimates are wide. This reflects a bias-variance tradeoff—to address potential confounding and improve the internal validity of our difference-in-differences estimates, we incorporated inverse probability of our treatment weights with event study models, which likely inflated the standard errors for our estimates. Indeed, a comparison of the weighted (Figure 3) and unweighted (S1_Figure) estimates supports this, as the magnitude of the effect estimates are similar, but the confidence intervals are wider for some outcomes in the weighted analyses. We have added a comment on this tradeoff to the limitations section of our Discussion. In addition, in accord with the Editor’s recommendations, we have removed causal language when referring to our estimates and indicate if the MHVS was, for example, positively or negatively associated with specific outcomes, along with the appropriate measure of association and 95% confidence interval.**

2. I feel these results are supportive of the continued rollout of the scheme, and of further analyses along similar lines in the future. This would be supported even more if there was data on the health economics of the scheme. Is that planned at some stage?

**We agree that an important next step would be to quantify the benefits of the program in terms of its costs. However, we have not conducted a detailed accounting of program costs, and this is not in our immediate plans.**

3. My only real question about the analysis comes from looking at Figure 2. I assume that we cannot read too much into these, as the MHVS is introduced at various times in different regions, and the curve for the "treated" line is a mixture of regions that have and have not yet received the scheme. However, the Institutional Delivery and Skilled Birth Attendant figures have a look of the intervention group lagging behind the control group, and catching up over time. This made me wonder whether the MHVS was delivered to regions partly on the basis of past/current health service utilization? If so, could some of the results simply represent regression to the mean? Were past levels of the outcomes of interest included as predictors in the propensity model used to derive the weights for the final analysis? Would this be an appropriate thing to do?

**We agree with the Reviewer that the trends in Figure 2 are for descriptive purposes and cannot be used to infer whether access to the MHVS, which started in 2006 in select upazilas and continued through 2010 (Figure 1), because the intervention was introduced at different times (an issue that is addressed by the event study models). In theory, the program was intended to roll-out to upazilas with priority given to socioeconomically disadvantaged areas based on literacy rates, poverty levels, and other indicators, as well as the availability of health workers. In practice, however, it was not geographically targeted based on poverty indicators.[1] Based on our descriptive analyses shown in Tables 2 and 3, and to the Reviewer’s point, it does seem that treated areas lagged behind control areas in terms of access to maternal health services (i.e., antenatal care, institutional delivery, skilled birth attendant), suggesting that there may have been some targeting based on health service utilization, but there were not clear differences in terms of stillbirth, neonatal mortality, or infant mortality.**

**Our goal was to account for measured differences between treated and control areas using inverse probability weights (see Appendix B, S2 Table, and S3 Table). We would have liked to include pre-intervention levels of the outcomes in our propensity model for the weights, but we could not identify a source of reliable upazila-level data on health service utilization, since they were not collected as part of the Census. Unfortunately, the DHS is not designed to provide representative estimates at the upazila-level. Thus, there is the potential for reverse confounding by past levels of our outcomes of interest, particularly for indicators of maternal health care use. This has been added as a limitation of our analyses.**

4. Tables 2 and 3 report the mean and standard deviation for various data items. This is unusual for categorical variables, as most of these are. I think most readers would feel more comfortable seeing these reported as frequencies and percentages.

**We appreciate this input and have revised Tables 2 and 3 so that means and SDs are reported for continuous variables and frequencies and percentages are reported for categorical variables.**

**Reviewer #2:**

This manuscript analyzes maternal service utilization and outcome data for delivery and newborn health across the implementation period of a national maternal health voucher system in Bangladesh. The authors have done an excellent job synthesizing the data and considered a range of potential scenarios in their sensitivity analysis and weighting approaches to counter

potential gaps in the data.

1. However, the most recent data included in this analysis is from 2014 while there was a DHS conducted in Bangladesh in 2018 - why were these data omitted from consideration as the MHVS appears to be an ongoing program?

**As mentioned in response to Query 8 from the Editor, our research protocol was completed in May 2019 and we began our analyses shortly thereafter, which was more than a year before the 2018 DHS were released by the DHS Program. Given how much effort goes into cleaning each wave, geocoding observations, assigning exposure/treatment, and completing our analyses, our relatively long study period of 14 years (2000-2013), with up to 7 years of post-intervention data, and the fact that few (n=2) upazilas joined the program between 2013 and 2018, we did not append the additional wave of data. Additionally, as noted by the Reviewer below, extending the study period by another 4-5 years could increase opportunities for unmeasured time-varying confounding by other local programs (see, for example, the study by Rahman et al. 2017[2]).**

2. Also, while I appreciate that the data are limited to those from DHS surveys, there is mention of the MHVS program not being implemented with fidelity in all areas or by all community health volunteers in the description of the program. How were these potential irregularities considered in the analysis? How might individual CHW variation in program implementation potentially impact findings?

**As indicated in Study Design section, we used a difference-in-differences design to estimate the intention-to-treat (ITT) effect of the MHVS, specifically the effect of providing upazila-level access to the program, which we consider the most policy relevant parameter. The ITT effect, which compares respondents based on their “treatment assigned”, is not biased by differences in fidelity across upazilas (e.g., CHW variation). However, ss we described in the third paragraph of the Discussion, prior literature suggests that that the implementation and targeting of the program was imperfect, which may have diluted the ITT effect compared to a different estimand, such as the complier average causal effect. Information on the implementation of the program within upazilas could be used to assess heterogeneous effects, which may be a next step for research on the MHVS, but these data were not publicly available. Additionally, if we had data on individual-level uptake of the program, it could be leveraged to estimate the impact of receiving a MHVS voucher on relevant outcomes; however, these data are not collected by the Bangladesh DHS. Thus, at this stage, we are estimating the ITT effect.**

3. Last, what other projects/programs were underway in Bangladesh through this period to either strengthen the supply side of the system or to further enhance patient demand? The analysis period is lengthy and in this time, there have been some notable shifts in care seeking and use in Bangladesh, principally for higher uptake of private services and a tremendous increase in Cesarean section rates. Could the authors please add these considerations to the Discussion in greater detail?

**We are happy to expand on these considerations in the Discussion. As we indicated in the revised Abstract and new Author Summary, we view changes to other health and social programs that might affect maternal health care use as the most plausible threats to the internal validity of our estimates. As the Reviewer indicated, there have been strong secular trends in selected indicators related to sexual, reproductive, maternal, and child health, which may be related to other health and social programs (see El Arifeen et al., 2013 for a summary[3]). A strength of the difference-in-differences design is that it accounts for secular trends that are common across treated and control areas. Thus, we would not expect confounding by large-scale changes such as the expansion of private providers or by smaller scale interventions unless they were correlated with the timing by which upazilas gained access to the MHVS. If, however, the existing stock of community health workers in each area influenced the process by which the MHVS was phased-in across upazilas and magnified its impact on health care services use, then the effect of providing access to the program may have been overestimated.**

4. In addition to these considerations, there is a minor revisions needed: Please change the word "unawareness" to "lack of awareness" or similar on the bottom of page 3.

**Thank you, this has been done.**

**Reviewer #3:**

This is an important evaluation of a longstanding voucher scheme in Bangladesh to promote maternal health and care seeking as well as birth outcomes. Its findings are broadly commensurate with other past evaluations in that there is a small increase in coverage (and hence likely care seeking) among recipients but that there are no tangible benefits on mortality.

Methodologically the authors did what was feasible in terms of identifying appropriate matched recipients and non-recipients for comparison.

1. One key point that needs further highlighting is if the sample size was sufficient for mortality estimation?

**We did not conduct a sample size or power analysis prior to our evaluation, primarily because the inferential goal of our observational study was to estimate the effect of providing access to the MHVS using existing (secondary) data. We endeavored to provide the least biased estimates of the impact of the MHVS along with a range of values that are compatible by that effect (represented by the 95% confidence interval). Although some of our estimates were relatively imprecise, as noted in our Discussion, we agree with the main argument of Hernán’s recent commentary, in which he stated, “It is preferable to have multiple studies with imprecise estimates than having no study at all.” Given the lack of available evidence on the relation between the MHVS and health outcomes, including stillbirth, neonatal mortality, and infant mortality, we are hopeful that our estimates are informative and gain relevance as further studies are conducted and evidence is synthesized.**

2. The key limitation in the analysis is the supply side, so acknowledged by the authors themselves. It would really help understand the findings if there were concomitant reasonable findings from the health systems for maternal and newborn health. These could be health workforce related, such as skilled birth attendants, facilities for transportation, newborn care etc. The distribution and coverage by CHWs or BRAC workers would be an additional variable. These data are available from other country analyses, and would help understand the quality of care issues that likely limited the benefits of this voucher scheme.

**We appreciate the Reviewer’s comment and agree that a process evaluation of the program, including the concomitant changes in supply-side factors, such as skilled birth attendants, helps to put our findings in context. In our Discussion, we have drawn on the best available evidence to comment on how the program was targeted and implemented, awareness of the program, enforcement of eligibility criteria, and changes in supply-side factors, such as enrollment of providers, provider remuneration, and the quality of available services.** [1, 4-7] **We agree that an upazila-level empirical evaluation of these process indicators would be valuable, but feel that it is outside the scope of our current study.**

**REFERENCES CITED**

1.	Khan MM, Khan AR. Report on the Diagnostic Study of Demand Side Financing—Maternal Health Voucher Scheme of Bangladesh. Dhaka, Bangladesh: Maxwell Stamp PLC; 2016.

2.	Rahman S, Choudhury AA, Khanam R, Moin SMI, Ahmed S, Begum N, et al. Effect of a package of integrated demand-and supply-side interventions on facility delivery rates in rural Bangladesh: implications for large-scale programs. PLoS One. 2017;12(10):e0186182.

3.	El Arifeen S, Christou A, Reichenbach L, Osman FA, Azad K, Islam KS, et al. Community-based approaches and partnerships: innovations in health-service delivery in Bangladesh. The Lancet. 2013;382(9909):2012-26.

4.	Hatt L, Nguyen H, Sloan N, Miner S, Magvanjav O, Sharma A, et al. Economic evaluation of demand-side financing (DSF) for maternal health in Bangladesh. Bethesda, Maryland: Review, Analysis and Assessment of Issues Related to Health Care Financing and Health Economics in Bangladesh, Abt Associates Inc; 2010.

5.	Ahmed S, Khan MM. A maternal health voucher scheme: what have we learned from the demand-side financing scheme in Bangladesh? Health policy and planning. 2011;26(1):25-32.

6.	Koehlmoos TLP, Ashraf A, Kabir H, Islam Z, Gazi R, Saha NC, et al. Rapid assessment of demand-side financing experiences in Bangladesh. Bangladesh: International Centre for Diarrhoeal Disease Research, Bangladesh; 2008.

7.	Talukder M, Rob U, Musa SAJM, Bajracharya A, Keya KT, Noor FR, et al. Evaluation of the impact of the voucher program for improving maternal health behavior and status in Bangladesh. Dhaka: Population Council; 2014.
