# Supplementary material for: Health care services use, stillbirth, and neonatal and infant survival following implementation of the Maternal Health Voucher Scheme in Bangladesh: A difference-in-differences analysis of Bangladesh Demographic and Health Survey data, 2000 to 2016
Source: PLoS Med. 2022 Aug 15;19(8):e1004022. doi: 10.1371/journal.pmed.1004022 (PMC9377610; doi:10.1371/journal.pmed.1004022)
Supplement: Attachment — Submitted filename: response to reviewers 20220331.docx [file pmed.1004022.s012.docx]

**REQUESTS FROM THE EDITORS:**

*“In light of the comments of Reviewer 3, we will only be able to consider a revised version which includes analysis of data from the most recent 2018 Bangladesh Demographic and Health Survey.”*

We have included data from the 2017-18 Bangladesh Demographic and Health Surveys and updated all methods, results, and corresponding tables and figures. Our results are qualitatively similar to our previous analyses, with no substantive changes to our main conclusions.

**COMMENTS FROM THE REVIEWERS:**

**Reviewer #1: Alex McConnachie, Statistical Review**

*“I am generally happy, though I still feel that the authors are overstating the results very slightly. The conclusion of the abstract states: "we found that the introduction of the MHVS was positively associated with the use of maternal health services". Whilst it looks likes this is probably the case, the fact remains that all three confidence intervals presented in support of this statement include zero. Ultimately, under the fitted models, the data are consistent with there being no associations. All I suggest is that this should be recognised more openly in the paper.”*

We appreciate the Reviewer’s comment. In our revised manuscript we have provided a more nuanced interpretation of our results in the abstract and results sections of the paper. We note, for example, that the introduction of the MHVS was associated with a robust increase in the probability of delivering at a health facility, and that the impacts on the receipt of antenatal care and skilled birth attendance were less apparent. For example, the abstract states:

“The introduction of the MHVS program was associated with a lagged improvement in the probability of delivering in a health facility, one of the primary targets of the program, although associations with other health services were less evident. Six years of access to the MHVS, the probabilities of reporting at least three antenatal care visits, delivering in a health facility, and having a skilled birth attendant present increased by 3.0 [95% confidence interval (95%CI)=-4.8, 10.7], 6.5 (95%CI=-0.6, 13.6), and 5.8 (95%CI=-1.8, 13.3) percentage-points, respectively.”

In the Results, the revised manuscript states, “There was less evidence for a sustained increase in the receipt of antenatal care; 6 years after program implementation, the probability of reporting at least three antenatal care visits increased by 3.0 (95%CI=-4.8, 10.7) percentage-points.”

**Reviewer #3:**

*“This is a revised version of a extensively reviewed study of the potential impact of a maternal health voucher scheme (MHVS) in Bangladesh on coverage and utilization of maternal health services between 2006 (the year of the introdyuction of the scheme) and 2013. The statistical methods for assessing lagged differences using a difference in differences approach are well described, even though the sample size was likely limited for a robust assessment of impact on mortality. 

The major limitation in the usefulness of this analysis for policy is lack of a longer term assesment of impact by inclusion of data from the recent most BDHS 2018 data and I found the arguments against including this latter period in the analysis, rather facile. inclusion of a longer time period of follow up could have answered key questions on program maturity and quality as well as secular trends. The policy relevance of data that are already almost a decade old is questionable.”*

As we indicated in our response to the Editor, we have updated all analyses after including the most recent data from the 2017-18 BDHS. The study period was extended by an additional three years (from 2013 to 2016). Our analyses now include observations on births that occurred up to 10 years after the implementation of the MHVS, and provide the longest-term follow-up and evaluation of this ongoing program.
